# Children’s sugar-sweetened beverages consumption: associations with family and home-related factors, differences within ethnic groups explored

**DOI:** 10.1186/s12889-017-4095-0

**Published:** 2017-02-14

**Authors:** V. M. van de Gaar, A. van Grieken, W. Jansen, H. Raat

**Affiliations:** 1 Department of Public Health, Erasmus University Medical Centre, Rotterdam, The Netherlands; 2 Department of Social Development, Rotterdam, The Netherlands

**Keywords:** Sugar-sweetened beverages, Child, Parenting, Home environment, Overweight prevention, Ethnic background

## Abstract

**Background:**

The consumption of sugar-sweetened beverages (SSB) may contribute to the development of overweight among children. The present study aimed to evaluate associations between family and home-related factors and children’s SSB consumption. We explored associations within ethnic background of the child.

**Methods:**

Cross-sectional data from the population-based ‘Water Campaign’ study were used. Parents (*n* = 644) of primary school children (6-13 years) completed a questionnaire on socio-demographic characteristics, family and home-related factors and child’s SSB intake. The family and home-related factors under study were: cognitive variables (e.g. parental attitude, subjective norm), environmental variables (e.g. availability of SSB, parenting practices), and habitual variables (e.g. habit strength, taste preference). Regression analyses were used to evaluate the associations between family and home-related factors and child’s SSB intake (*p* < 0.05).

**Results:**

Mean age of the children was 9.4 years (SD: 1.8) and 54.1% were girls. The child’s average SSB intake was 0.9 litres (SD: 0.6) per day. Child’s age, parents’ subjective norm, parenting practices, and parental modelling were positively associated with the child’s SSB intake. The availability of SSB at home and school and parental attitude were negatively associated with the child’s SSB intake. The associations under study differed according to the child’s ethnic background, with the explained variance of the full models ranging from 8.7% for children from Moroccan or Turkish ethnic background to 44.4% for children with Dutch ethnic background.

**Conclusions:**

Our results provide support for interventions targeting children’s SSB intake focussing on the identified family and home-related factors, with active participation of parents. Also, the relationships between these factors and the child’s SSB intake differed for children with distinct ethnic backgrounds. Therefore, we would recommend to tailor interventions taking into account the ethnic background of the family.

**Trial registration:**

Number NTR3400; date April 4th 2012; retrospectively registered.

**Electronic supplementary material:**

The online version of this article (doi:10.1186/s12889-017-4095-0) contains supplementary material, which is available to authorized users.

## Background

Consumption of sugar-sweetened beverages (SSB) among primary school children has been found to be positively associated with obesity and other health problems later in life [1–6]. To increase the effectiveness of interventions aiming to decrease children’s SSB intake, it is necessary to identify the factors underlying their SSB consumption [7–9].

Cognitive models [10, 11] assist in providing an understanding of how behaviours such as SSB consumption develop as a result of cognitive factors such as attitude (i.e. behavioural beliefs), subjective norm (i.e. perceived social pressure) and perceived behavioural control (i.e. perceived ease or difficulty of performing the behaviour) [12, 13]. However, combined behavioural and environmental models like for instance the Environmental Research framework for weight Gain prevention (EnRG-framework) [14], indicate that physical or social environmental factors also influence health behaviours [15–18]. Parents, and consequently the home environment, play an essential role in establishing healthy behaviours for children [19–22]. Therefore, factors of interest include availability and accessibility of SSB at home, food rules and parenting practices (i.e. specific behaviours that parents use while/to raising their children), as well as parental involvement and role modelling [22, 23]. Additionally, it has been argued that habit strength (i.e. how strong a learned behavioural response is to a situational cue) and taste preferences (i.e. liking one food over another) are considered important factors to understand health behaviours [24–26]. Thus far, studies have yielded mixed results with regard to the associations between family and home-related factors and the child’s SSB consumption [23, 27–36].

Overweight prevalence between children with certain ethnic backgrounds differs [37–40]. Consequently, awareness has been raised about potential differences regarding factors determining the child’s SSB intake among families and children with different ethnic backgrounds [41–43]. More specifically, eating culture and parenting styles differ for families with diverse ethnic backgrounds [44, 45]. In addition, previous research shows that families from different ethnic minority groups may live in more (or less) obesogenic home environments, which subsequently may influence their healthy and unhealthy behaviours with respect to other demographic groups [46–50]. Brug and colleagues described that eating culture in relation to the child’s food environment differs across countries in Europe and that it is recommended to implement different strategies to improve healthy behaviours among children [43]. For instance, a study found that Dutch children consumed the most SSB compared to other European countries and that the SSB consumption of children living in countries with less SSB-friendly environments was lower [40].

Studies specifically focussing on evaluating the association between ethnic background (within one region) and family and home-related factors are lacking [46, 47], and non-existing within the Dutch multi-ethnic setting within the larger cities. According to Wyse and colleagues, their research among Australian preschool children showed the social-cultural environment (e.g. family eating patterns) as the most amendable to intervene [51]. Deepening our understanding of the associations within different ethnic groups will improve the ability to develop culturally appropriate interventions [52]. Based on their systematic review, Gubbels and colleagues emphasize that the next step within lifestyle research should be to differentiate and tailor interventions according to moderating factors described in the EnRG-framework to enhance interventions’ effectiveness [53].

Therefore, insights into and understanding of the associations between family and home-related factors with child’s health behaviour within different ethnic groups may contribute to the development of these interventions tailored to specific subgroups. In this study, the objective was to first study associations between family and home-related factors with child’s SSB intake and second, to explore these associations within different ethnic groups. Our hypothesis was: the associations between family and home-related factors with child’s SSB intake are different within distinct ethnic groups.

## Methods

### Study population and procedure

Our cross-sectional study used data from the population-based ‘Water Campaign’ study [54]. This controlled trial assessed the effects of a combined school- and community-based intervention on children’s SSB consumption. The Medical and Ethical Review Committee of the Erasmus Medical Centre issued a ‘declaration of no objection’ (i.e. formal waver) for this study (reference number MEC-2011-183). Four primary schools located in multi-ethnic neighbourhoods in Rotterdam, the Netherlands, were included in the study; two schools were included as intervention schools, two schools were included as control schools. Intervention and control schools were matched on number of pupils, socio-economic status and overweight prevalence. The included schools resulted from a convenience sample of schools participating in a municipal overweight intervention program. Only schools in disadvantaged neighbourhoods were eligible for this intervention [54].

All children of grades 2 to 8 (aged 6 to 13 years) within each of the four included schools were invited to participate, resulting in a total of 1288 invited children. Passive parental consent was obtained. Parents (and children) received a brochure with information to notify and inform them about the intervention and study participation. The study was also announced by the school, via the school letter and through the teachers and by flyers which were visible throughout the school. Parents (and children) were free to refuse participation without giving any explanation. They could do so by informing one of the teachers at their school or one of the researchers when present at school. At all times, the researchers could be contacted by a special phone-number or e-mail, for instance to decline participation [54]. Measurements were performed at baseline and after one year, using questionnaires (child and parental) and observations at school.

For the present study, data from the baseline parental questionnaire (administered March/April 2011) was used. A study population of 644 children (6 to 13 years) was available for analyses.

## Measures

### Socio-demographic characteristics child and parent

The child’s gender (boy/girl), age (years), and ethnic background were assessed. Ethnic background was defined by country of birth of the parents according to definitions given by Statistics Netherlands [55]. The child’s ethnic background was defined as Dutch only if both parents had been born in the Netherlands; if one of the parents had been born in another country, ethnic background was defined according to that country; and if both parents had been born in different foreign countries, ethnic background was defined as the mother’s country of birth. Ethnic background was categorized as ‘Dutch’; ‘Surinamese/Antillean’; ‘Moroccan/Turkish’; or ‘other/unknown’.

Respondents were either the father or the mother of the child, and parental gender was based on this item (male/female). From this point onwards, respondent is described as ‘parent’. Parental age (years) and educational level were also reported. Based on standard Dutch cut-off points, parents’ highest achieved educational level was categorized as ‘low’ (no education; primary school; ≤3 years of general secondary school); ‘mid-low’ (>3 years of general secondary school); ‘mid-high’ (higher vocational training; undergraduate programs); or ‘high’ (higher academic education) [56].

In addition to the parental questionnaire, weight and height of the child were obtained by trained personnel. Weight status was determined by calculating BMI in kg/m2 with height measured to the nearest 0.1 cm and weight measured to the nearest 0.2 kg, in light clothing or gym clothes, according to a national standardized protocol for Youth Health Care [57]. Children were categorized as being either ‘non-overweight’ or ‘overweight/obese’, based on cut-off points published by the International Obesity Task Force [58].

### Family and home-related factors

Additional file 1: Table S1 provides an overview of the scales (and items) that were used to measure the family and home-related factors: (a) cognitive variables, (b) environmental variables, and (c) habitual variables. The measures of the cognitive variables and the environmental variables were based on the studies ‘ENDORSE’ and ‘Be Active, Eat Right’ [59, 60]. They were developed using the Theory of Planned Behaviour (TPB) as proposed by Ajzen [10]. The items were tailored to the consumption of SSB by children as suggested by Oluka et al. [61] and by Francis et al. [62]. The items used to measure the construct ‘habit’ were derived from the Self-Report Behavioural Automaticity index [63, 64].

In Additional file 1: Table S1 we report the percentages of missing answers per item and per scale as indicator of the feasibility of the measurements in our study. Scales were only computed when there were no missing data on any of the items. Additional file 1: Table S1 also shows the Cronbach’s alpha per scale to assess the internal consistency of each multi-item scale.

The cognitive variables assessed were parental attitude towards the child’s SSB consumption (two items, Cronbach’s α = 0.84), parental attitude towards decreasing the child’s SSB consumption (four items, Cronbach’s α = 0.70), parents’ subjective norm towards the child’s SSB consumption (one item), and the perceived behavioural control of parents towards having their child drink less SSB (two items, Cronbach’s α = 0.75). The percentage of missing values ranges from 2.6% to 7.6%; the internal consistency measured by Cronbach’s alpha is ≥0.7 for two multi-item scales and >0.8 for one scale (all scales show ‘good’ internal consistency [65]).

The environmental variables assessed included the availability of SSB at home and school (two items, Cronbach’s α = 0.64), parenting practices towards the child’s SSB intake (four items, Cronbach’s α = 0.74), rules at home with regard to the child’s SSB intake (two items, Cronbach’s α = 0.77), and modelling of SSB consumption by the parents (one single item; and a two items-scale, Cronbach’s α = 0.72). The percentage of missing values ranges from 2.3% to 6.2%; the internal consistency measured by Cronbach’s alpha is >0.7 for three of the multi-item scales (which is considered ‘good’), and >0.6 for one scale (which is considered ‘moderate’ [65]).

The habitual variables assessed were habit strength of the child’s SSB intake (four items, Cronbach’s α = 0.76) and taste preference of the child towards SSB (one item). The percentage of missing values ranges from 1.6% to 4.7%; the internal consistency measured by Cronbach’s alpha is >0.7 for the multi-item scale (which is considered ‘good’ [65]).

All items measuring the family and home-related factors were assessed by using a five-point response scale, except for the questions regarding restriction rules for the child’s SSB consumption (response scale ‘yes’/‘no’). All items were coded such that a higher score indicated more unfavourable behaviour (i.e. the child was expected to consume more SSB). The internal consistency of the multi-item scales was overall ‘moderate’ to ‘good’ (Cronbach’s α > 0.60) [65, 66]. The relatively low number of missing values supports the feasibility of the measures. We recommend further research regarding the validity of these measurement instruments in diverse populations.

### SSB consumption

The following definition of SSB was used: beverages containing added sugar, sweetened dairy products (e.g. chocolate milk), fruit juices (e.g. apple juice), soft drinks (e.g. cola) and energy drinks (e.g. sport energy drinks). Examples of SSB were provided along with the question based on our definition of SSB.

Average SSB consumption was assessed using the question ‘On a day your child drinks SSB, how many glasses (250 ml), cans (330 ml) or bottles (500 ml) does your child consume on average?’. Response categories ranged from ‘none’ to ‘5 or more’. The child’s average SSB intake in litre per day was calculated by multiplying each reported glass, can and/or bottle with its volume and summed up thereafter.

### Statistical analysis

Child and parental characteristics were analysed using descriptive statistics. Linear regression models were fitted. The dependent variable was the child’s average SSB intake in litre per day. The family and home-related factors (cognitive, environmental and habitual variables) of SSB consumption were used as independent variables in the model.

The independent variables were entered in the model as blocks, correcting for other variables within this block. The first block that was entered in the model contained the socio-demographic characteristics (child’s age, gender and ethnic background, and parental educational level); the second block contained the cognitive variables (parental attitude, parents’ subjective norm, and perceived behavioural control); the third block contained the environmental variables (availability, parenting practices, rules, and parental modelling); and the fourth and final block contained the habitual variables (habit strength and child’s taste preference). Finally, a full model was fitted with all independent variables. Beta’s with 95% confidence intervals (95% CI) were estimated. The (adjusted) R square is presented to indicate the estimated amount of explained variance for each model. Results were considered significant at *p* < 0.05 [66].

To explore differences in family and home-related factors according to ethnic background of the child, an interaction term was added to the model. Separately per block of variables (cognitive, environmental, and habitual) an interaction between ethnic background and the variable of interest was analysed, the model being only corrected for the variables in that block and not for any other (socio-demographic) variables. All interaction analyses are presented in Additional file 2: Table S2; several interactions differed statistically (*p* < 0.10) [66]. Therefore, the previously mentioned full model was fitted separately for the subgroups of children with a Dutch, Surinamese/Antillean, Moroccan/Turkish, and other/unknown ethnic background.

Analyses were conducted using SPSS version 22.0 (IBM Corp., Armonk, NY, USA).

## Results

### Study population characteristics

Mean age of the children was 9.4 years (SD: 1.8), 54.1% were girls, 30.3% were Dutch and 23.0% were overweight or obese. Parents’ mean age was 37.0 years (SD: 8.9), 87.4% were female and 18.5% indicated to have completed a high level of education. According to the parents, the average SSB intake of the children was 0.9 litres per day (SD: 0.6) per day (Table 1).

**Table 1:** Child and parental characteristics for the overall sample and according to ethnic background of the child (*n* = 644)

	Overall sample (*n* = 644) % or mean (SD)	Dutch (*n* = 195) % or mean (SD)	Surinamese/Antillean (*n* = 142) % or mean (SD)	Moroccan/Turkish (*n* = 185) % or mean (SD)	Other/unknown (*n* = 119) % or mean (SD)	*p*-value^a^
*CHILD characteristics*						
Gender, % girl *missing, n = 12*	54.1%	55.2%	53.9%	50.0%	58.8%	0.500
Age (in years), mean (SD) missing, *n* = 6	9.4 (1.8)	8.7 (1.8)	9.4 (1.8)	9.6 (1.6)	10.4 (1.6)	**0.000**
Ethnic background						
% Dutch				30.3%		
% Surinamese/Antillean				22.0%		
% Moroccan/Turkish				28.9%		
% Other/unknown				18.8%		
Weight status, % overweight or obese missing, *n* = 45	23.0%	13.8%	26.1%	31.8%	21.1%	**0.001**
*PARENTAL characteristics*						
Gender, % female missing, *n* = 47	87.4%	88.8%	94.8%	82.4%	84.0%	**0.007**
Age (in years), mean (SD) missing, *n* = 5	37.0 (8.9)	37.3 (8.6)	36.7 (7.7)	36.4 (9.4)	37.6 (10.0)	0.655
Educational level missing, *n* = 21						**0.000**
% Low		22.0%	10.6%	11.4%	41.2%	23.2%
% Mid-low		25.0%	30.7%	23.6%	25.3%	17.0%
% Mid-high		34.5%	32.3%	47.1%	24.7%	38.4%
% High		18.5%	26.5%	17.9%	8.8%	21.4%
*SSB intake child*						
Average SSB in litre per day, mean (SD) missing, *n* = 3	0.9 (0.6)	0.7 (0.4)	1.1 (0.9)	0.8 (0.5)	0.9 (0.5)	**0.000**

^a^Differences between groups stratified for outcome measures, tested with one-way Anova (continuous variables) and Chi-square test (categorical variables)

Note: Numbers printed in bold represent significant differences between the ethnic backgrounds groups

Table 1 also shows the differences in socio-demographic variables and child’s SSB intake for children from different ethnic backgrounds. For instance, differences were found between children with a Dutch ethnic background and children with a Surinamese or Antillean ethnic background with regard to average SSB intake: 0.7 litres per day (SD: 0.4) for Dutch children vs. 1.1 litres per day (SD: 0.9) for Surinamese or Antillean children (*p* < 0.05).

### Associations related to the child’s SSB intake

Table 2 presents the results of the regression analyses evaluating the full model. The explained variance of the full model was 25.9% of the child’s SSB intake.

**Table 2:** Results from the linear regression models evaluating the associations between family and home related factors and child’s SSB intake in litre per day

	Model 1 (*n* = 625)	Model 2 (*n* = 570)	Model 3 (*n* = 565)	Model 4 (*n* = 611)	Model 5 (*n* = 527)
	beta (95% CI)	beta (95% CI)	beta (95% CI)	beta (95% CI)	beta (95% CI)
*Socio-demographic characteristics*					
Gender child, boy = ref	-0.05 (-0.14; 0.04)				-0.02 (-0.10; 0.06)
Age child (in years)	0.05 (0.02; 0.08)***				0.05 (0.02; 0.07)***
Ethnic background child					
Dutch	- REF -				- REF -
Surinamese/Antillean	0.32 (0.19; 0.44)***				0.20 (0.08; 0.31)**
Moroccan/Turkish	-0.02 (-0.15; 0.10)				-0.01 (-0.12; 0.10)
Other/unknown	0.01 (-0.13; 0.15)				0.01 (-0.11; 0.14)
Educational level of parent					
Low	0.15 (0.01; 0.29)*				0.01 (-0.12; 0.15)
Mid-low	-0.08 (-0.21; 0.06)				-0.05 (-0.17; 0.07)
Mid-high	0.15 (0.03; 0.28)*				0.08 (-0.04; 0.19)
High	- REF -				- REF -
*Cognitive variables* ^*a*^					
Attitude		0.14 (0.06; 0.21)***			0.04 (-0.04; 0.12)
Attitude towards decreasing SSB		-0.07 (-0.12; -0.01)*			-0.08 (-0.14; -0.02)**
Subjective norm		0.16 (0.11; 0.21)***			0.09 (0.04; 0.15)***
Perceived behavioural control		0.05 (0.00; 0.10)*			-0.00 (-0.05; 0.05)
*Environmental variables* ^*a*^					
Availability			-0.03 (-0.06; 0.01)		-0.04 (-0.08; -0.01)*
Parenting practices			0.21 (0.13; 0.28)***		0.13 (0.05; 0.20)**
Rules			0.09 (-0.02; 0.20)		0.06 (-0.04; 0.17)
Modelling			0.11 (0.07; 0.15)***		0.06 (0.02; 0.10)**
−Modelling *separate item*			0.01 (-0.02; 0.04)		0.02 (-0.01; 0.05)
*Habitual variables* ^*a*^					
Habit strength				0.20 (0.15; 0.25)***	0.05 (-0.01; 0.11)
Taste preference				0.01 (-0.04; 0.06)	0.01 (-0.04; 0.05)
R2 (adjusted)^b^	**.095**	**.127**	**.163**	**.101**	**.259**

*REF* reference category

^a^Higher scores indicate expectation of more SSB consumption/higher score on unfavourable behaviour

^b^R square statistic represents the estimated level of variance explained by the regression model

Note: numbers printed in bold represent significant association between independent variable and average SSB consumption in litre per day of child in that model. Asterisks’ represent the level of significance of the association between independent variable and outcome, corrected for all other variables: *p < 0.05, **p < 0.01, ***p < 0.001

Child’s SSB intake associations according to ethnic background of the child

Presented in Table 3 are the full models of the child’s average SSB intake in litre per day separately for the four subgroups based on ethnic background. The explained variance of the full models ranged from 8.7% for children from Moroccan or Turkish ethnic background to 44.4% for children with a Dutch ethnic background.

**Table 3:** Results from the full linear regression model evaluating the associations between family and home related factors and child’s SSB intake in litre per day according to the ethnic background of the child

	Dutch (*n* = 169)	Surinamese/Antillean (*n* = 112)	Moroccan/Turkish (*n* = 151)	Other/unknown *(n* = 95)
	beta (95% CI)	beta (95% CI)	beta (95% CI)	beta (95% CI)
*Socio-demographic characteristics*				
Gender child, boy = ref	-0.06 (-0.16; 0.03)	0.14 (-0.13; 0.41)	-0.09 (-0.23; 0.05)	-0.05 (-0.23; 0.13)
Age child (in years)	0.03 (0.00; 0.05)*	0.07 (-0.00; 0.15)	0.04 (-0.00; 0.08)	0.08 (0.03; 0.14)**
Educational level of parent				
Low	0.14 (-0.03; 0.31)	-0.09 (-0.62; 0.43)	0.03 (-0.20; 0.26)	0.09 (-0.18; 0.37)
Mid-low	-0.08 (-0.20; 0.05)	-0.09 (-0.49; 0.31)	-0.08 (-0.31; 0.16)	0.12 (-0.18; 0.41)
Mid-high	0.09 (-0.03; 0.22)	0.07 (-0.28; 0.42)	0.08 (-0.16; 0.32)	0.10 (-0.15; 0.34)
High	- REF -	- REF -	- REF -	- REF -
*Cognitive variables* ^*a*^				
Attitude	0.13 (0.03; 0.24)*	0.04 (-0.27; 0.34)	-0.02 (-0.15; 0.12)	0.14 (-0.04; 0.32)
Attitude towards decreasing SSB	0.00 (-0.07; 0.08)	-0.25 (-0.44; -0.05)*	-0.01 (-0.13; 0.10)	-0.00 (-0.13; 0.12)
Subjective norm	0.06 (-0.02; 0.12)	0.19 (-0.01; 0.38)	0.04 (-0.03; 0.12)	0.14 (0.02; 0.26)*
Perceived behavioural control	-0.05 (-0.10; 0.01)	0.03 (-0.16; 0.22)	0.03 (-0.06; 0.13)	-0.03 (-0.14; 0.08)
*Environmental variables* ^*a*^				
Availability	0.01 (-0.04; 0.05)	-0.05 (-0.21; 0.10)	-0.07 (-0.13; -0.02)*	-0.00 (-0.10; 0.09)
Parenting practices	0.09 (0.01; 0.18)*	0.26 (-0.01; 0.52)	0.10 (-0.04; 0.23)	-0.08 (-0.25; 0.10)
Rules	-0.06 (-0.19; 0.07)	0.15 (-0.19; 0.48)	-0.12 (-0.31; 0.07)	0.39 (0.17; 0.60)**
Modelling	0.05 (0.01; 0.09)*	0.07 (-0.09; 0.22)	0.08 (0.01; 0.15)*	0.09 (-0.00; 0.18)
− Modelling *separate item*	0.03 (0.00; 0.06)*	0.06 (-0.02; 0.15)	-0.03 (-0.09; 0.03)	0.01 (-0.06; 0.07)
*Habitual variables* ^*a*^				
Habit strength	0.11 (0.05; 0.17)**	0.01 (-0.21; 0.23)	0.00 (-0.11; 0.11)	0.06 (-0.11; 0.22)
Taste preference	0.01 (-0.05; 0.06)	-0.02 (-0.17; 0.14)	0.01 (-0.07; 0.10)	-0.09 (-0.20; 0.03)
R2 (adjusted)^b^	**.444**	**.278**	**.087**	**.249**

*REF* reference category

^a^Higher scores indicate expectation of more SSB consumption/higher score on unfavourable behaviour

^b^R square statistic represents the estimated level of variance explained by the regression model

Note: Results from the full model with all independent variables; numbers printed in bold represent significant association between independent variable and average SSB consumption in litre per day of child. Asterisks’ represent the level of significance of the association between independent variable and outcome, corrected for all other variables: *p <  0.05, **p <  0.01

The results are reported in accordance with STROBE (Strengthening the Reporting of Observational Studies in Epidemiology) [67].

## Discussion

In this paper, we evaluated associations between family and home-related factors and children’s SSB intake. Overall, child’s age, parental attitude, parents’ subjective norm, the availability of SSB at home and school, parenting practices and parental modelling showed to be associated with child’s average SSB intake in litre per day. Associations between family and home-related factors and child’s SSB intake differed for children with a Dutch, Surinamese/Antillean, Moroccan/Turkish, and other/unknown background.

In line with previous studies among children of similar age, we observed that children of parents who have a more positive attitude towards decreasing the child’s SSB intake or children of parents with a more positive subjective norm towards their child’s SSB intake, consumed less SSB [24, 28, 31, 59]. Also, children of parents who express healthier parenting practices towards the child’s SSB intake (i.e. more restrictive towards the child’s SSB consumption) and children of parents who less often model SSB consumption , are reported to have a lower SSB intake [18, 34, 41, 68, 69].

Contrary to other studies, we found that children consume less SSB when there is more SSB available in the home or school environment [29, 31, 33, 69]. This contradiction could be due to the cross-sectional nature of our study; children who already have high consumption levels might have less SSB available, following already implemented restrictions of their parents trying to improve the child’s lifestyle.

The significant positive associations between parenting practices and parental modelling and the child’s SSB consumption, emphasize the important role of parents in shaping the child’s dietary habits [17, 20, 21, 33, 34, 52, 68]. Parents serve both as role model and as facilitator impacting children’s consumption diet. To increase interventions’ effectiveness, parents should be involved or specifically targeted as intervention participants [22, 36, 43, 68].

In this paper, we explored whether the associations between the family and home-related factors under study and the child’s SSB intake differed according to ethnic background of the child. As Verzeletti emphasized, ethnic background differences may have an impact on parental beliefs regarding the child’s SSB consumption or on rules restricting the intake of SSB by the child [41]. Our results provide support for this statement.

Children with a Dutch ethnic background showed positive associations between the child’s SSB intake and parental attitude, parenting practices and parental modelling. Contrary to the model for all children together, our findings suggest that habit strength is an important determinant (only) for children with a Dutch ethnic background. Our model explained the most (44%) of the child’s SSB intake for children with a Dutch ethnic background.

For children with a Surinamese or Antillean ethnic background, parental attitude towards decreasing the child’s SSB intake was the only determinant that showed a significant association with the child’s SSB consumption; the model explained almost 28% of the child’s SSB consumption. We recommend future studies to explore factors that better explain the child’s SSB consumption among this specific group. In the meantime, in order to decrease the child’s SSB intake, special attention should be given to parental attitude when targeting children from Surinamese or Antillean ethnic background. For instance, health education as intervention element may be suggested as an element to change attitudes among these families [70].

Our model could explain just 9% of the child’s SSB intake for children with a Turkish or Moroccan ethnic background. Given this small percentage of explained variance, we recommend future research to further explore and identify factors associated with child’s SSB consumption. The two significant associations of factors with SSB consumption among Turkish or Moroccan children that were observed in the model appeared to be in the home environment (e.g. availability and modelling). Our results suggest that for children with a Turkish or Moroccan ethnic background, intervening on family level may be beneficial in order to reduce SSB consumption and not only improve the child’s lifestyle but also that of the family. Especially with regard to the availability of SSB, the family (e.g. parents) are responsible for buying SSB and determine to what extent SSB is provided to their child. Also parents’ modelling behaviour could be addressed in these family level interventions, for instance by including skills training and role play in the intervention [70].

For children with an ‘other or unknown’ ethnic background, the model explained almost 25%. However, interpretation of the results is difficult because of the varied composition of children in this group. It included for example children with non-Western (e.g. Afghanistan) and Western background (e.g. Germany). We have no explanation for the association between child’s age, parents’ subjective norm and rules at home and the child’s SSB intake.

Our results provide support for differences in the association between family and home-related factors and the child’s SSB intake according to the ethnic background of the child. Further research is needed to increase our understanding of these differences. It has been suggested that associations between demographic factors and health-related behaviours as were found in this study, might be due to differences in health literacy [71]. How well people understand and can act on health-related information has shown to be associated with healthy lifestyles [72]. Caregiver’s health literacy has been associated with their own and their children’s’ health outcomes [73–77]. Increased understanding of these factors and underlying mechanisms that possibly can explain the children’s lifestyle behaviours between subgroups could assist to further tailor and improve interventions, in order to enhance interventions’ effectiveness.

### Methodological considerations

Evaluating the child’s SSB intake by means of a continuous measure, average consumption in litre per day is considered a strength of this study. Because of the possibility of information loss, using a continuous measure is preferred above transforming the measure into a dichotomous variable. However, seen by other studies, SSB intake is often reported dichotomously as ≤2 vs >2 SSB servings per day. When conducting the analyses with child’s intake in ≤2 vs >2 SSB servings per day as outcome measure, we observed similar associations between family and home-related factors and child’s SSB intake (both for the overall analyses as when exploring between children with different ethnic backgrounds; see Additional file 3: Tables S3-S6). The diverse population with children from various ethnic backgrounds and a response of 54.8% on the parent questionnaire (given the diverse population) are also strengths that have to be mentioned.

However, some limitations of the present study need to be addressed. We relied on parental self-reports, which is a commonly used way to assess children’s intake. Though there is a possibility that parents may have provided socially desirable answers, parent reports are seen as one of the most accurate methods to estimate a child’s intake (in the ages 4 to 11 years old) [78]. Children’s weight and height were measured by trained health professionals, applying a standardised protocol for Youth Health Care [57]. The measurements of family and home-related factors were based on the TPB and tailored to the consumption of SSB by children, as suggested by Oluka et al. [61] and Francis et al. [62]. In our study, the Cronbach’s alphas indicate ‘moderate’ to ‘good’ reliability of the multi-item scales. We recommend further research regarding the validity of these measurement instruments in multi-ethnic populations. Given the cross-sectional design of our study, inferences regarding cause and effect are not possible. It is recommended to explore and test our findings for causal inferences in longitudinal or experimental intervention studies. Also, the study was conducted in multi-ethnic inner-city neighbourhoods. The generalizability of our study findings might therefore be limited to children belonging to similar populations and settings.

## Conclusions

This paper provided insight into factors related to children’s SSB consumption. We observed that the child’s age, parental attitude, parents’ subjective norm, the availability of SSB at home and school, parenting practices, parental modelling were associated with the child’s SSB consumption (in litre per day). These findings provide support for interventions to focus on parents and improve their (family) lifestyle in order to promote the transference of healthy behaviours to the children.

Moreover, we observed differences with respect to the associations between family and home-related factors and child’s SSB consumption for children with a Dutch, Surinamese/Antillean, Moroccan/Turkish, and other/unknown background. Therefore, further understanding of the factors of health behaviour of different target segments should be endorsed, through observational, qualitative research and quantitative research as well as longitudinal studies replicating our findings. By identifying the most important factors per target segment intervention effectiveness may be improved. In the meantime, we recommend intervention developers and behaviour change agents in the field to take relevant differences into account when developing tailored interventions within multi-ethnic communities.

## Additional files

Additional file 1: Table S1. Descriptive results and scale information for the family and home-related factors (*n* = 644). (PDF 142 kb)
Additional file 2: Table S2.
*P*-values for interaction between ethnic background and the determinants on child’s SSB intake in litre per day (*n* = 644). (PDF 219 kb)
Additional file 3: Tables S3-S6.
**Table S3.** SSB intake in servings per day for the overall sample and according to ethnic background of the child (*n* = 644). **Table S4.** *P*-values for interaction between ethnic background and the determinants on child’s SSB intake in servings per day (*n* = 644). **Table S5.** Results from the logistic regression models evaluating the associations between family and home-related factors and child’s SSB intake in servings per day (≤2 vs >2). **Table S6.** Results from the full logistic regression model evaluating the associations between family and home-related factors and child’s SSB intake in servings per day (≤2 vs >2) according to ethnic background child. (PDF 281 kb)

## Abbreviations

BMI: Body Mass Index
EnRG-framework: Environmental Research framework for weight Gain prevention
SSB: sugar-sweetened beverages
TPB: Theory of Planned Behaviour
