# Supplementary material for: Children’s sugar-sweetened beverages consumption: associations with family and home-related factors, differences within ethnic groups explored
Source: BMC Public Health. 2017 Feb 14;17:195. doi: 10.1186/s12889-017-4095-0 (PMC5310003; doi:10.1186/s12889-017-4095-0)
Supplement: Additional file 1: Table S1. — Descriptive results and scale information for the family and home-related factors (n = 644). (PDF 142 kb) [file 12889_2017_4095_MOESM1_ESM.pdf]

Table S1. Descriptive results and scale information for the family and home-related factors (n=644)

	General information		Scale information		(Example of) Questionnaire item
	n (%)	mean (SD)	# items	Cronbach's α (stand.)	
Cognitive variables[1]					
Parental attitude towards child's SSB intake *(range 1-5)*	625 (97.0%)	3.0 (0.6)	2	0.8	e.g. "When my child drinks SSB, I find it…" (pleasant – not so pleasant)
Parental attitude towards decreasing child's SSB intake *(range 1-5)*	595 (92.4%)	2.2 (0.8)	4	0.7	e.g. "I believe my child should consume less SSB." (agree – disagree)
Parents subjective norm towards the child's SSB intake *(range 1-5)*	627 (97.4%)	2.3 (0.9)	1	-	i.e. "When comparing your child with other children of his/her age, does your child consume more or less SSB?" (more – less)
Perceived behavioural control of parents towards having their child drink less SSB *(range 1-5)*	625 (97.0%)	2.3 (0.9)	2	0.8	e.g. "Does it seem difficult or easy to let your child drink less SSB?" (difficult – easy)
Environmental variables[1]					
Availability of SSB at home/school *(range 1-5)*	629 (97.7%)	3.7 (1.3)	2	0.6	e.g. "SSB's are usually available for my child at home." (agree – disagree)
Parenting practices towards child's SSB intake *(range 1-5)*	625 (97.0%)	2.7 (0.7)	4	0.7	e.g. "To what extent do you monitor how often your child drinks SSB?" (never – always)
Rules at home with regard to child's SSB intake *(range 1-2)*	624 (96.9%)	1.4 (0.4)	2	0.8	e.g. "Are there in your home rules about how many SSB your child may consume?" (yes – no)
Modelling of SBB intake by the parents *(range 1-5)*	617 (95.8%)	2.6 (1.2)	2	0.7	e.g. "How often do you (or your partner) drink SSB together with your child? (never – every day, multiple times)
– Separate item 'Parental Modelling' *(range 1-5)*	604 (93.8%)	2.5 (1.5)	1	-	i.e. "Does your partner consume SSB often?" (never – always)

Habitual variables[1]					
Habit strength of the child's SSB intake *(range 1-5)*	614 (95.3%)	3.0 (0.9)	4	0.8	e.g. "My child often drinks SSB without thinking about it." (agree – disagree)
Taste preference of child towards SSB *(range 1-5)*	634 (98.4%)	4.3 (1.0)	1	-	i.e. "My child likes the taste of SSB." (agree – disagree)

[1]Higher scores indicate the expectation of more SSB consumption/a higher score on unfavourable behaviour.
