# Supplementary material for: Children’s sugar-sweetened beverages consumption: associations with family and home-related factors, differences within ethnic groups explored
Source: BMC Public Health. 2017 Feb 14;17:195. doi: 10.1186/s12889-017-4095-0 (PMC5310003; doi:10.1186/s12889-017-4095-0)
Supplement: Additional file 2: Table S2. — P-values for interaction between ethnic background and the determinants on child’s SSB intake in litre per day (n = 644). (PDF 219 kb) [file 12889_2017_4095_MOESM2_ESM.pdf]

Table S2. *P*-values for interaction between ethnic background and the determinants on child's SSB intake in litre per day* (n=644)

	SSB in litre		
	p-value<br>Dutch vs Surinamese/Antillean	*p*-value<br>Dutch vs Moroccan/Turkish	*p*-value<br>Dutch vs Other/unknown
***Cognitive variables*[1]**			
Parental attitude towards child's SSB intake *(range 1-5)*	*NS*	0.003	*NS*
Parental attitude towards decreasing child's SSB intake *(range 1-5)*	0.002	*NS*	*NS*
Parents subjective norm towards the child's SSB intake *(range 1-5)*	0.009	*NS*	*NS*
Perceived behavioural control of parents towards having their child drink less SSB *(range 1-5)*	0.002	*NS*	*NS*
***Environmental variables*[1]**			
Availability of SSB ate home/school *(range 1-5)*	*NS*	0.014	*NS*
Parenting practices towards child's SSB intake *(range 1-5)*	<0.001	*NS*	*NS*
Rules at home with regard to child's SSB intake *(range 1-2)*	0.001	*NS*	0.007
Modelling of SBB intake by the parents *(range 1-5)*	0.073	*NS*	*NS*
– Separate item 'Parental Modelling' *(range 1-5)*	*NS*	*NS*	*NS*
***Habitual variables*[1]**			
Habit strength of the child's SSB intake *(range 1-5)*	0.059	0.011	*NS*
Taste preference of child towards SSB *(range 1-5)*	*NS*	*NS*	*NS*

*Separately per block of variables (cognitive, environmental, and habitual) the interaction was analysed tested with one-way Anova (continuous variables) and Chi-square test (categorical variables), being only corrected for the variables in that block and not for any other variables or socio-demographic variables. As seen in this supplement table 2, several interactions differed statistically (p<0.10) [60].

NS = not significant (*p*>0.10).

[1]Higher scores indicate the expectation of more SSB consumption/a higher score on unfavourable behaviour.
