# Supplementary material for: Children’s sugar-sweetened beverages consumption: associations with family and home-related factors, differences within ethnic groups explored
Source: BMC Public Health. 2017 Feb 14;17:195. doi: 10.1186/s12889-017-4095-0 (PMC5310003; doi:10.1186/s12889-017-4095-0)
Supplement: Additional file 3: Tables S3-S6. — Table S3. SSB intake in servings per day for the overall sample and according to ethnic background of the child (n = 644). Table S4. P-values for interaction between ethnic background and the determinants on child’s SSB intake in servings per day (n = 644). Table S5. Results from the logistic regression models evaluating the associations between family and home-related factors and child’s SSB intake in servings per day (≤2 vs >2). Table S6. Results from the full logistic regression model evaluating the associations between family and home-related factors and child’s SSB intake in servings per day (≤2 vs >2) according to ethnic background child. (PDF 281 kb) [file 12889_2017_4095_MOESM3_ESM.pdf]

Table S3. SSB intake in servings per day for the overall sample and according to ethnic background of the child (n=644)

	Overall sample *(n=644)* % or mean (SD)	**Dutch** (n=195) % or mean (SD)	**Surinamese/Antillean** (n=142) % or mean (SD)	**Moroccan/Turkish** (n=185) % or mean (SD)	**Other/unknown** (n=119) % or mean (SD)	*p*-value[a]
SSB intake child						
>2 SSB servings per day, % yes	59.3%	54.9%	71.8%	55.7%	57.1%	**0.007**

a = differences between groups stratified for outcome measures, tested with one-way Anova (continuous variables) and Chi-square test (categorical variables).
Note: numbers printed in **bold** represent significant differences between the ethnic backgrounds groups.

Table S4. ***P*****-values for interaction between ethnic background and the determinants on child's SSB intake in servings per day* (n=644)**

	SSB servings		
	p-value Dutch vs Surinamese/Antillean	*p*-value Dutch vs Moroccan/Turkish	*p*-value Dutch vs Other/unknown
Cognitive variables[1]			
Parental attitude towards child's SSB intake *(range 1-5)*	0.002	0.003	0.011
Parental attitude towards decreasing child's SSB intake *(range 1-5)*	*NS*	*NS*	*NS*
Parents subjective norm towards the child's SSB intake *(range 1-5)*	*NS*	*NS*	*NS*
Perceived behavioural control of parents towards having their child drink less SSB *(range 1-5)*	*NS*	*NS*	*NS*
Environmental variables[1]			
Availability of SSB ate home/school *(range 1-5)*	*NS*	0.022	0.080
Parenting practices towards child's SSB intake *(range 1-5)*	*NS*	*NS*	*NS*
Rules at home with regard to child's SSB intake *(range 1-2)*	*NS*	*NS*	0.032
Modelling of SBB intake by the parents *(range 1-5)*	*NS*	0.066	0.065
– Separate item 'Parental Modelling' *(range 1-5)*	0.088	*NS*	*NS*
Habitual variables[1]			
Habit strength of the child's SSB intake *(range 1-5)*	*NS*	<0.001	*NS*
Taste preference of child towards SSB *(range 1-5)*	*NS*	*NS*	*NS*

*Separately per block of variables (cognitive, environmental, and habitual) the interaction was analysed, being only corrected for the variables in that block and not for any other variables or socio-demographic variables. As seen in this supplement table 4, several interactions differed statistically (p<0.10) [60].
NS = not significant (p>0.10).
[1]Higher scores indicate the expectation of more SSB consumption/a higher score on unfavourable behaviour.

Table S5. Results from the logistic regression models evaluating the associations between family and home-related factors and child's SSB intake in servings per day (≤2 vs >2)

	Model 1 (n=604)	Model 2 (n=570)	Model 3 (n=565)	Model 4 (n=611)	Model 5 (n=516)
	OR (95%CI)	OR (95%CI)	OR (95%CI)	OR (95%CI)	OR (95%CI)
Socio-demographic characteristics					
Gender child, boy=ref	**0.65 (0.46;0.92)***				0.75 (0.49;1.14)
Age child (in years)	**1.21 (1.09;1.34)*****				**1.27 (1.11;1.44)*****
Ethnic background child					
Dutch	- REF -				- REF -
Surinamese/Antillean	1.52 (0.93;2.50)				1.11 (0.60;2.05)
Moroccan/Turkish	**0.61 (0.38;0.98)***				0.61 (0.34;1.10)
Other/unknown	0.64 (0.37;1.09)				0.55 (0.28;1.07)
Educational level of parent					
Low	**3.30 (1.87;5.83)*****				**2.20 (1.10;4.42)***
Mid-low	1.30 (0.78;2.15)				1.23 (0.66;2.30)
Mid-high	**3.10 (1.89;5.08)*****				**2.31 (1.26;4.22)****
High	- REF -				- REF -
Cognitive variables[1]					
Attitude		**1.48 (1.08;2.04)***			0.74 (0.48;1.14)
Attitude towards decreasing SSB		0.84 (0.66;1.06)			0.93 (0.69;1.26)
Subjective norm		**1.96 (1.55;2.48)*****			**1.62 (1.22;2.14)****
Perceived behavioural control		1.04 (0.84;1.27)			0.80 (0.60;1.05)
Environmental variables[1]					
Availability			1.04 (0.90;1.21)		0.98 (0.81;1.19)
Parenting practices			**1.84 (1.32;2.56)*****		**1.54 (1.04;2.28)***
Rules			1.60 (0.98;2.60)		1.31 (0.75;2.29)
Modelling			**1.78 (1.47;2.14)*****		**1.60 (1.29;1.98)*****
– Modelling *separate item*			0.90 (0.79;1.03)		0.94 (0.81;1.09)
Habitual variables[1]					
Habit strength				**2.08 (1.68;2.56)*****	**1.49 (1.08;2.06)***
Taste preference				0.92 (0.76;1.13)	1.01 (0.79;1.30)
**Nagelkerke R^2 (adjusted)**[2]	**.133**	**.126**	**.222**	**.121**	**.330**

REF = reference category.
[1] Higher scores indicate expectation of more SSB consumption/higher score on unfavourable behaviour.
[2] Nagelkerke R square statistic represents the estimated level of variance explained by the regression model.
Note: numbers printed in **bold** represent significant association between independent variable and **≤2 vs >2 SSB intake in servings per day** of child in that model. Asterisks' represent the level of significance of the association between independent variable and outcome, corrected for all other variables: * p<0.05, ** p<0.01, *** p<0.001.

Table S6: Results from the full logistic regression model evaluating the associations between family and home-related factors and child's SSB intake in servings per day (≤2 vs >2) according to ethnic background child

	**Dutch** (n=167)	**Surinamese/Antillean** (n=111)	**Moroccan/Turkish** (n=147)	**Other/unknown** (n=91)
	OR (95%CI)	OR (95%CI)	OR (95%CI)	OR (95%CI)
Socio-demographic characteristics				
Gender child, boy=ref	**0.37 (0.14;0.97)***	1.20 (0.35;4.09)	**0.41 (0.18;0.94)***	1.17 (0.40;3.39)
Age child (in years)	1.20 (0.92;1.56)	1.34 (0.94;1.91)	**1.35 (1.06;1.73)***	1.37 (0.96;1.96)
Educational level of parent				
Low	**8.67 (1.37;54.79)***	5.83 (0.47;71.84)	0.98 (0.21;4.50)	0.99 (0.19;5.25)
Mid-low	0.46 (0.13;1.63)	5.36 (0.90;31.96)	0.63 (0.14;2.94)	1.43 (0.26;7.68)
Mid-high	3.09 (0.93;10.28)	**5.35 (1.22;23.42)***	0.80 (0.16;3.97)	1.76 (0.39;7.99)
High	- REF -	- REF -	- REF -	- REF -
***Cognitive variables*[1]**				
Attitude	2.41 (0.78;7.51)	**0.13 (0.03;0.56)****	1.05 (0.50;2.23)	0.99 (0.34;2.90)
Attitude towards decreasing SSB	1.11 (0.55;2.26)	0.55 (0.23;1.29)	1.07 (0.56;2.05)	1.03 (0.49;2.16)
Subjective norm	1.85 (0.91;3.77)	1.26 (0.53;3.02)	**1.95 (1.20;3.16)****	1.25 (0.59;2.66)
Perceived behavioural control	0.82 (0.45;1.48)	1.24 (0.55;2.80)	0.79 (0.45;1.41)	0.80 (0.42;1.54)
***Environmental variables*[1]**				
Availability	1.15 (0.74;1.79)	1.60 (0.84;3.03)	0.87 (0.62;1.22)	0.76 (0.43;1.34)
Parenting practices	1.72 (0.74;4.02)	2.78 (0.92;0.84)	**2.18 (1.00;4.75)***	0.90 (0.32;2.54)
Rules	0.76 (0.20;2.83)	0.97 (0.23;4.15)	1.12 (0.37;3.40)	**5.56 (1.43;21.69)***
Modelling	**2.18 (1.39;3.42)****	**2.67 (1.30;5.49)****	1.34 (0.87;2.08)	1.54 (0.91;2.62)
– Modelling *separate item*	1.33 (0.97;1.84)	0.73 (0.48;1.12)	0.78 (0.55;1.10)	1.09 (0.73;1.62)
***Habit variables*[1]**				
Habit strength	**2.50 (1.31;4.77)****	1.61 (0.64;4.08)	0.68 (0.36;1.28)	2.38 (0.89;6.37)
Taste preference	0.69 (0.35;1.32)	0.95 (0.51;1.76)	1.17 (0.72;1.91)	0.80 (0.40;1.57)
**Nagelkerke R^2 (adjusted)[2]**	**.592**	**.495**	**.281**	**.356**

REF = reference category.
[1] Higher scores indicate expectation of more SSB consumption/higher score on unfavourable behaviour.
[2] Nagelkerke R square statistic represents the estimated level of variance explained by the regression model.
Note: results from the full model with all independent variables; numbers printed in **bold** represent significant association between independent variable and **≤2 vs >2 SSB intake in servings per day** of child in that model. Asterisks' represent the level of significance of the association between independent variable and outcome, corrected for all other variables: * p<0.05, ** p<0.01, *** p<0.001.
